# *Aggregatibacter actinomycetemcomitans* (*Aa*) Under the Radar: Myths and Misunderstandings of *Aa* and Its Role in Aggressive Periodontitis

**DOI:** 10.3389/fimmu.2019.00728

**Published:** 2019-04-16

**Authors:** Daniel H. Fine, Amey G. Patil, Senthil K. Velusamy

**Affiliations:** Department of Oral Biology, Rutgers School of Dental Medicine, Newark, NJ, United States

**Keywords:** *A. actinomycetemcomitans*, leukotoxin, habitat, nutritional sustenance, biogeographical mobilization, aggressive periodontitis

## Abstract

*Aggregatibacter actinomycetemcomitans (Aa)* is a low-abundance Gram-negative oral pathobiont that is highly associated with a silent but aggressive orphan disease that results in periodontitis and tooth loss in adolescents of African heritage. For the most part *Aa* conducts its business by utilizing strategies allowing it to conceal itself below the radar of the host mucosal immune defense system. A great deal of misinformation has been conveyed with respect to *Aa* biology in health and disease. The purpose of this review is to present misconceptions about *Aa* and the strategies that it uses to colonize, survive, and evade the host. In the process *Aa* manages to undermine host mucosal defenses and contribute to disease initiation. This review will present clinical observational, molecular, and interventional studies that illustrate genetic, phenotypic, and biogeographical tactics that have been recently clarified and demonstrate how *Aa* survives and suppresses host mucosal defenses to take part in disease pathogenesis. At one point in time *Aa* was considered to be the causative agent of Localized Aggressive Periodontitis. Currently, it is most accurate to look at *Aa* as a community activist and necessary partner of a pathogenic consortium that suppresses the initial host response so as to encourage overgrowth of its partners. The data for *Aa's* activist role stems from molecular genetic studies complemented by experimental animal investigations that demonstrate how *Aa* establishes a habitat (housing), nutritional sustenance in that habitat (food), and biogeographical mobilization and/or relocation from its initial habitat (transportation). In this manner *Aa* can transfer to a protected but vulnerable domain (pocket or sulcus) where its community activism is most useful. *Aa*'s “strategy” includes obtaining housing, food, and transportation at no cost to its partners challenging the economic theory that “there ain't no such thing as a free lunch.” This “strategy” illustrates how co-evolution can promote *Aa's* survival, on one hand, and overgrowth of community members, on the other, which can result in local host dysbiosis and susceptibility to infection.

## Introduction

Ever since 1976 when it was discovered that *Aggregatibacter actinomycetemcomitans* (ne Actinobacillus) was associated with Aggressive Periodontitis in adolescents there have been many attempts to understand its relationship to disease (1, 2). *A. actinomycetemcomitans* (*Aa*) was first reported by Klinger in 1912 where he described a previously unknown Gram-negative microorganism that was found in actinomycotic lesions associated with Actinomyces, hence the latin word “comitans” in common with Actinomyces (3). In addition to its association with aggressive periodontitis, *Aa* has been implicated as an organism associated with a variety of systemic diseases including but not limited to; infectious endocarditis, brain abscesses, and chest wall abscesses (4). While initially it was thought that *Aa* was the cause of localized aggressive periodontitis (LAgP) (5) current research suggests that *Aa* is implicated as an important and perhaps necessary constituent of a consortium of microorganisms related to disease (6). What follows is a review that focuses on major trends that have supported, and in some cases misrepresented the role of *Aa* in the LAgP disease process. The review will divide *Aa*'s role in the disease process into several steps that are required for this specific microorganism to participate in an infection that attacks the periodontal attachment apparatus and bone. In this review the disease provoking process has been divided into four steps as follows; Step 1: colonization above the gum-line, Step 2: integration and survival in the biofilm milieu, Step 3: migration to a new setting below the gumline, and Step 4: suppression of the mucosal host defenses below the gum-line. Many of these steps have been clarified in recent years by harmonizing; (a) clinical observational studies in humans (7), (b) studies using molecular approaches (8), and (c) interventional/experimental studies in animal models (9). As a result it is now possible to put forward a narrative that illustrates how *Aa* can actively participate in the disease process.

## Misconception 1: *Aa* is a Late Colonizer

### Clinical

In seminal experiments in the mid-1960's it became clear that dental plaque/biofilm formation is due to synchronized events that begin with deposition of salivary proteins above the gumline on the native tooth surface, followed by accumulation of Streptococcal species onto the glycoprotein layer set down on the enamel (10). In the first 2-days following plaque deposition on the tooth surface streptococcal species can amount to up to 90% of the tooth related microbiota followed by actinomyces species. These pioneer colonizers form parallel arrays, perpendicular to the tooth surface interspersed by lactate utilizing Veillonella (11). Over a 3-week period the composition of plaque changes from a predominantly Gram-positive aerobic Streptococcal microbiota to a mixed Gram positive and Gram negative facultatively anaerobic flora containing streptococci, *Actinomyces* sp, *Veillonella* sp, *Fusobacteria* sp, vibrios, spirochetes, and others (12).

Early on there were controversies related to *Aa*'s attachment properties (8). These disputes were due to the fact that ATCC laboratory strains were used in early studies that focused on *Aa* attachment (13). When these lab strains were investigated they failed to demonstrate the natural aggregative tendency of *Aa*, and thus *Aa* was shown to adhere poorly (13). This concept was re-inforced by Kolenbrander and associates who studied co-aggregation and suggested that *Aa* was a poor colonizer since, the ATCC strain Y4 only coaggregated with *Fusobacteria nucleatum*, the universal coaggregator (14, 15). These coaggregation/microbial interactions have, with the exception of *Aa*, been shown, for the most part, to play a critical role in plaque chronology (15). Thus, it was suggested that *Aa* was a late colonizer incapable of participation in early plaque formation (16).

### Molecular

**Figure 1:**
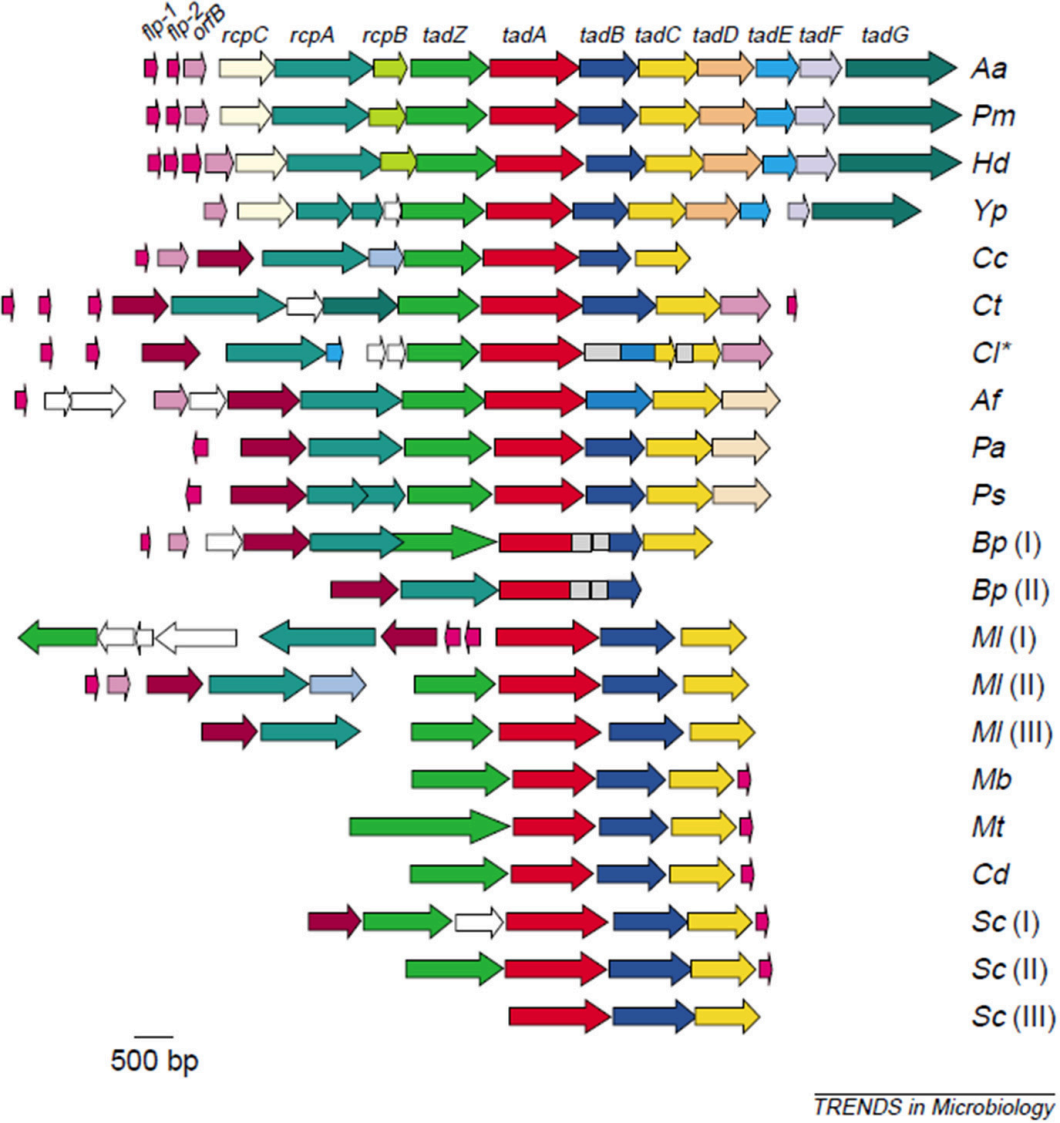
Illustration of the widespread colonization island (WCI). WCI consists of a 14 gene operon seen in its complete genetic composition in *(Aa) Aggregatibacter actinomycetemcomitans, (Pm)Pasteurella multocida, (Hd) Haemophilus ducreyi*. Remainder of microbes have a portion of the WCI: *(Yp)Yersinia pestis. (Cc) Caulobacter crescentus, (Ct) Chlorobium tepidum, (Cl) Chlorobium limicola, (Af) Acidithiobacillus ferooxidans, (Pa) Pseudomonas aeruginosa, (Ps) P. syringae, (Bp) Bordetella pertussis* [two clusters], *(MI) Mesorhizobium loti* [three clusters], *(Mb) Mycobacterium bovis, (Mt) M. tuberculosis, (Cd) Corynebacterium diphtheriae, (Sc) Streptomyces coelicolor* [three custers]. This island is responsible for binding to abiotic surfaces and is present in many pathobionts and in all Archae sequenced to date (22).

The first evidence to strengthen support for *Aa*'s adherence properties were shown by comparing attachment of laboratory strains to clinical isolates derived from the same parental strain (17). Lessons learned from these comparisons led to an understanding of how to maintain the clinical adherent phenotype in the laboratory, which led to the discovery of the Widespread Colonization Island (WCI) (13, 18). The WCI discovered in 2001 consists of a 14 gene operon and was shown to contain the *flp, tad*, and *rcp* genes that are intimately related to attachment to abiotic surfaces, aggregation, and tight adherence (18–20). This genomic island was present in many pathogenic strains including; *Yersinia pestis, Haemophilus ducreyi, Psuedomonas aeroginosa, Bordetella pertussis, Caulobacter crescentus, Salmonella enterica, Eschericia coli*, and all Archea sequenced to date (19). The discovery of the WCI undoubtedly influenced the change in the genus name from *Actinobacillus* to *Aggregatibacter* and demonstrated how important attachment was for survival of even the most primitive species (21). The fact that so many pathobionts contain the functional portion of this island attest to the importance of adherence in their persistence (Figure 1).

The specificity of *Aa*'s binding was illustrated by the fact that *Aa* obtained from pre-dentate children showed a high degree of tissue and species specificity (23, 24). In addition, *Aa* strains from parent and child often showed the same genotype demonstrating patterns of vertical transmission (25).

The studies described above were further supported by the discovery of an outer membrane adhesin, Aae, which showed specificity for oral epithelium (26). Unlike the WCI, Aae bound to its receptor on buccal epithelial cells (BECs) in a highly specific, dose dependent manner, that was saturable and thus achieved a plateau where no further binding was attainable (27). This data coupled with the finding that the Aae adhesin showed selective binding to BECs obtained from humans and old world primates but did not bind to BECs obtained from dogs, cats, pigs, goats etc., demonstrated a high level of species and tissue specificity (28). Further, subsequent reports revealed that *Aa* moved from tissue to teeth after teeth erupted depicting the direction of movement within specific oral domains (28, 29).

In a clear contrast to the non-specificity of the binding to abiotic surfaces via the WCI, binding of *Aa* via outer membrane adhesins were shown to be highly specific. *Aa* therefore has the capacity for both specific and non-specific mechanisms of adherence in the oral cavity.

### Experimental

The difficulty in changing the initial perception that *Aa* was a poor colonizer was due to the fact that it was hard to demonstrate that *Aa* was seen in the early stages of plaque formation. Clinical proof that *Aa* was a hardy colonizer was elusive because; (1) *Aa* was rarely found in plaque isolated from healthy individuals (5, 7), and (2) when *Aa* was isolated from periodontally diseased individuals it aggregated (13, 20). After isolation from clinical sites, *Aa* had a characteristic rough colonial texture that clumped on subculture and was highly adherent to abiotic surfaces. However, after repeated passage in the laboratory *Aa* reverted to a smooth colonial morphology that did not clump and was minimally adherent (13) (see Figure 1) This rough to smooth conversion fit the description of phase variation demonstrated by several pathogens that change from rough to smooth (*Neisseria*) enhancing their pathogenic strategy (30). However, in studies of *Aa*, this shift was seen *in-vitro* (12) but was not seen *in-vivo* in animal models and thus has been proposed to be a laboratory artifact (31).

**Figure 2:**
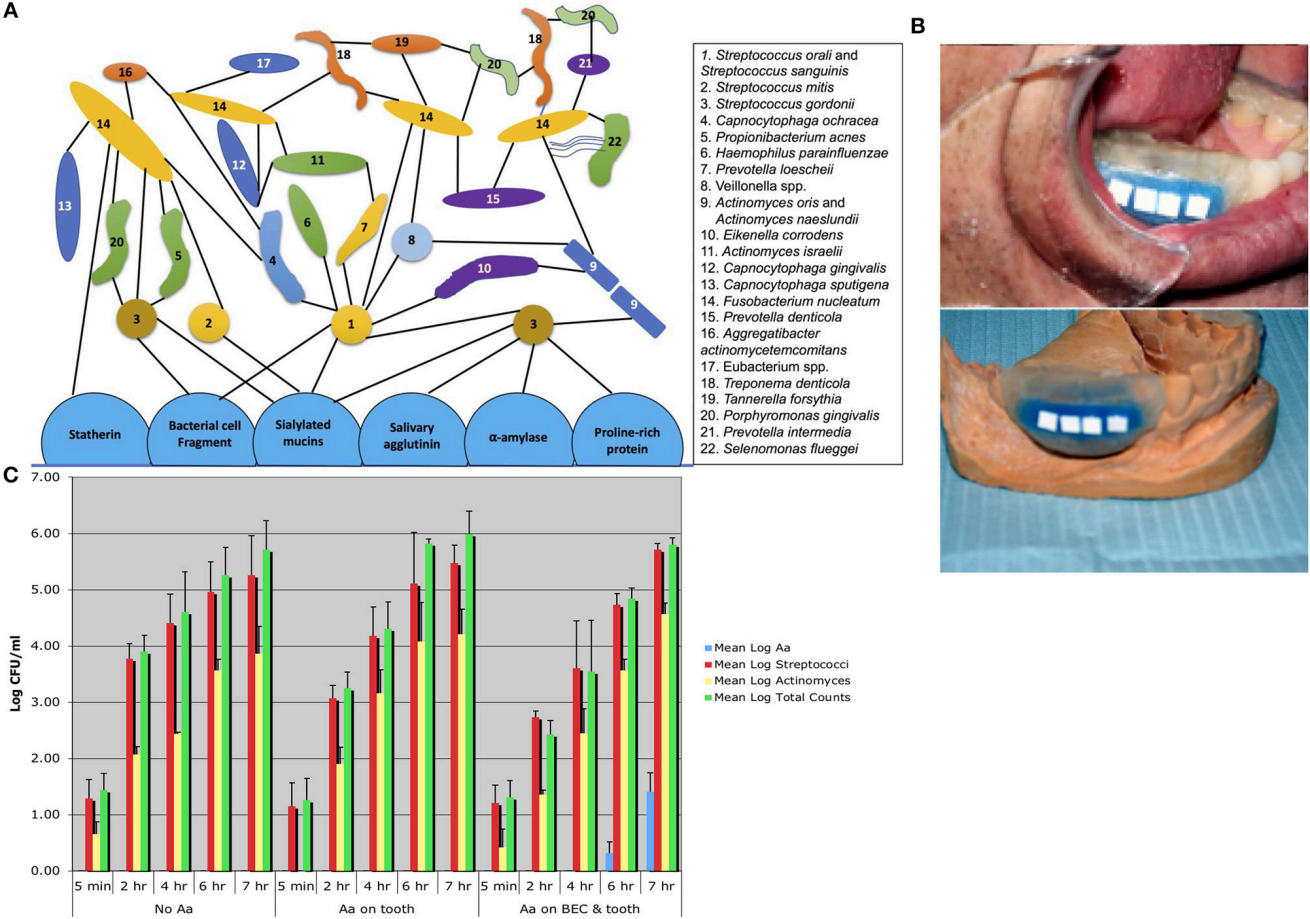
Diagram of plaque biofilm topography and chronology contrasted to *in-vivo* studies illustrating *Aa* colonization. Diagram shows pioneer colonizers closest to tooth surface and late colonizers on outer border of plaque geography [Adpated from Kolenbrander et al. (16)]. Note that *Aa* #16 in this figure is depicted as a late colonizer attached to *Fusobacterium nucleatum* **(A)**. Picture of stent fabricated for human volunteers containing Hydroxyapatite (HA) squares used for the capture of oral microorganisms that show early binding in subjects who harbor *Aa* **(B)**. Bar graph showing three panels 5 min to 7 h. First panel on left represents bacteria found over the 7 h period in subjects who had no *Aa* (controls). Middle panel shows subjects who had *Aa* on tooth surface only where no *Aa* is found on HA squares. Panel on right shows subjects with *Aa* on buccal cells and teeth, where *Aa* has migrated from buccal cells to HA squares and is detected on square at 6 and 7 h time-point **(C)** [Adapted from Fine et al. (32)].

To characterize *Aa* in humans it was necessary to survey over 100 adolescents in order to select 5 individuals who had *Aa* on buccal epithelial cells (BECs) and on tooth surfaces, and 5 individuals who had *Aa* only on tooth surfaces, as compared to 3 individuals who did not have *Aa* in their oral cavity (32). Stents were fabricated for these subjects and hydroxyapatite (HA) squares or discs were inserted into the stent. The stents were placed into the mouths of these subjects and HA squares were removed 5 min, 1, 4, 6, and 7 h after placement. Following HA square removal the square was sonicated, and the bacteria removed from the HA square after sonication were plated for determination of total bacteria, *streptococcal* spp., *Actinomyces* spp and *Aa* colonies (Figure 2). In these experiments subjects who had *Aa* on their BECs, were found to have *Aa* on HA squares within 6 h after the start of the *in-vivo* experiment, however none of those with *Aa* on teeth alone showed *Aa* on the HA squares (32). In a parallel series of *in-vitro* experiments, pre-treatment of BECs with *Aa* where shown to have been colonized by *Aa*. Reaction of these *Aa* colonized BECs with sterile/untreated HA squares in a test tube showed that *Aa* migrated and attached to the HA squares *in-vitro*. In contrast, HA squares pre-treated with *Aa* showed that *Aa* attached avidly to HA squares *in-vitro*. However, when these HA treated squares were reacted with untreated/uncontaminated BECs in a test tube, *Aa* attached to HA did not migrate to BECs. Results showed that while *Aa* was incapable of moving from HA to BECs, *Aa* was quite capable of moving from BECs to HA. These *in-vivo* and *in-vitro* experiments clearly demonstrated the difference in the kinetics of *Aa* attached to BECs via specific adhesin/receptor interactions that are reversible, as compared to linear and irreversible, non-specific aggregative adherence mediated by *flp, tad*, and *rcp* genes that facilitate *Aa's* attachment to HA (32).

Confirmation of the early plaque formation of *Aa* came from a little known experiment conducted in 1976 by Kilian and Rolla where these investigators set out to study the dietary impact of sucrose on *Streptococcus mutans* colonization in a Rhesus monkey model (33). In these experiments it was clearly shown that sucrose influenced early colonization of *S. mutans*, but unintentionally it was also demonstrated that *Aa*, a member of the commensal flora in these monkeys, was found on teeth within 3 h following thorough oral debridement of these Rh monkeys.

### Conclusions

Current data indicates that *Aa* can colonize tooth surfaces above the gum-line and that this is an early event. In the section below we will describe how *Aa* can compete for nutritional elements with a multitude of rival bacteria in order to survive.

## Misconception 2: Nutritional Fastidious Nature of *Aa*

### Clinical

*Aa* has been characterized as a fastidious microbe that requires CO_2_, serum, and specific carbohydrates such as glucose for its growth (34). It has been reported that *Aa* has a mandatory requirement for 5% CO_2_ for primary isolation, and that its growth is purported to be boosted by addition of serum (34). The difficult recovery of the microbe from potentially diseased sites throughout the body has been attributed to *Aa's* **f**astidious nature and its slow and inconsistent growth after initial isolation. These characteristics have suggested that the organism is difficult to isolate from human sites and difficult to grow in the laboratory. These features coupled with the aggregative nature of the microbe when grown in the laboratory has made quantitative assessment of the microbe problematic. Further, these features have discouraged a more intense investigation of the genetic traits of *Aa* (13). More recently genetic methodologies have made it easier to do quantitative assessment of the microbe from individual periodontal sites of interest.

### Molecular

In 2007, Brown and Whiteley demonstrated that *Aa* preferentially metabolizes lactate over other more readily available carbon sources such as glucose and fructose due to novel exclusion methods controlled by phosphoenol pyruvate-dependent phosphtranferase systems (35). Reduced levels of lactate appeared to limit *Aa*'s ability to grow and in the presence of high glucose consuming competitors such as Streptococci, *Aa* was shown to thrive. It was proposed that *Aa*'s survival was due to reduced competition with fast-growing and abundant members of the pioneer plaque flora such as Streptococci that utilized glucose as their carbon source. Further proof was obtained by creation of a lactic acid dehydrogenase deficient strain of *Aa* that fully ablated *Aa* growth (36). Results were also supported by the fact that lactate added to chemically defined media and/or the addition of lactate producing Streptococci caused increased growth of *Aa* in multi-community co-culture biofilms.

Moreover, Stacy et al. (37) showed that the toxic elements (H_2_O_2_) produced by co-culture with *S. gordonii* were overcome by the up-regulation *katA, apiA*, and *dspB* genes that protect *Aa* from the destructive effects of H_2_O_2_. These *in-vitro* reductionist models (and *in-vivo* dual species mouse studies) have been substantiated in human and primate models that examined both supra and sub gingival plaque (38).

### Experimental

**Figure 3:**
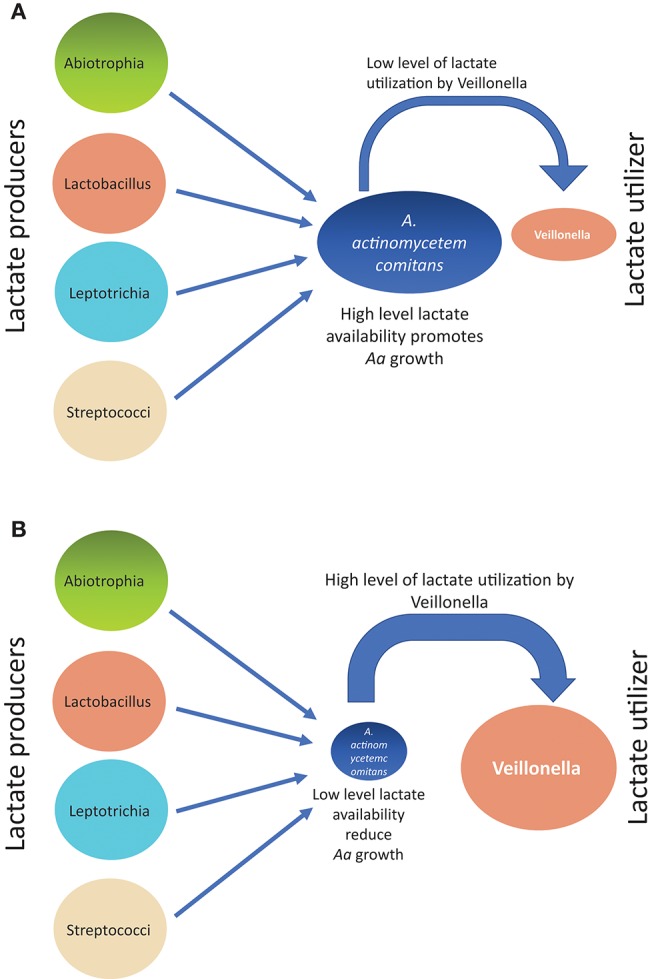
Diagram illustrating how growth of *Aa* can be influenced from associations of lactate producers (LP) as compared to lactate utilizers(LU) in Rh primate plaque studies. Panel on top **(A)** illustrates the scenario describing relative *Aa* growth when there is a low level of lactate utilization by Veillonella. Panel on bottom **(B)** illustrates the scenario describing relative *Aa* growth when there is a high level of lactate utilization by Veillonella leaving a low level of lactate available for *Aa* consumption.

Supragingival plaque communities were examined in an interventional/experimental Rhesus (Rh) primate model (9). With respect to *Aa*, Rh primates are unique in that the overwhelming majority of healthy primates harbor *Aa* in their supragingival plaque (9). This consistent presence of *Aa* in supragingival plaque allows for examination of *Aa*'s role in early plaque formation (9). By using a modification of a specific correlation analysis (39) we were able to substantiate and extend the observations of the Whiteley group (38). This network analysis allowed us to select the top 50 microbes by means of operational taxonomic units via 16S RNA identification. Selecting *Aa* as the node in the analysis of the 16 Rh monkeys with fully formed plaque biofilms, we were able to show that several key lactate producing species including streptococci*, Leptotrichia*, and *Abiotrophia* as well as *Veillonella* (a microbe with a strict lactate requirement) were highly associated with the hyper-leukotoxin producing strain of *Aa* (Table 1). This was true at baseline and 4-weeks after plaque reformed after thorough debridement (40). Calculation of lactate availability favored the growth and survival of *Aa* in a real-world environment [(39); Figure 3].

**Table 1:** Estimation of lactate availability comparing Veillonella to lactate producers.

	**Wild Type- RhAa3**			**RhAa-ltx P530**		
	**Wk 1**	**Wk 2**	**Wk 4**	**Wk 1**	**Wk 2**	**Wk 4**
**Genus**	**OTUs**	**OTUs**	**OTUs**	**OTUs**	**OTUs**	**OTUs**
Abiotrophia	3,915	1,662	12,778	3,136	627	2,654
Leptotrichia	227	2,422	6,002	147	1,915	863
Streptococci	3,255	18,750	70,261	7,359	14,658	6,591
Total: Producers (P)	7,397	22,834	89,001	10,642	17,200	10,108
Veillonella- Utilizers (U)	3,718	13,689	35,861	1,355	3,395	381
Ratio: P/U	2/1	1.67/1	2.5/1	7.85/1	5.1/1	26.5/1

*OTUs: Operational Taxonomic Units; Wk: Week; Ratio: Producers/Utilizers*.

### Conclusion

Molecular and experimental Rh primate models support the concept that *Aa* preferentially associates with lactate producing species which enables *Aa* to survive by limiting its competition for glucose as a carbon source used by other pioneer colonizers. The Rh primate model support and extend the findings of the Whiteley group in a real world competitive natural model.

## Misconception 3: A Highly Aggregative Non-motile Microbe Cannot Escape From its Biofilm Habitat

### Clinical

After a great deal of research it became obvious that *Aa* aggregates avidly in its “native” state. This dramatic phenotype led to the question as to how *Aa* could be trapped in one location and migrate to another. Without this capability to migrate, *Aa* would have to be designated as a pathobiont with biogeographically limited capabilities (13).

The importance of the transfer of *Aa* from a position above the gum-line to the cul de sac shaped sulcus below the gum-line is critical for transition from health to disease. The subgingival habitat differs significantly from the domain seen above the gum-line. The subgingival domain is anaerobic, filled with a serum transudate derived from vessels contained in the subjacent connective tissue. The junctional epithelium (JE) forms the base of the sulcus and borders the tooth surface. The “V” shaped structure of the sulcus surrounds the tooth. The left side of the V is the junctional epithelium (JE) derived from reduced enamel epithelium, remnants of the enamel organ (41), while the right side of the V is sulcular epithelium derived from oral epithelial tissue precursors.

These JE cells are one to two layers thick and are extremely permeable to outward flow of serum and cells. Directly beneath the basement membrane of the JE is a dense vascular network (42). Within 3–4 days after abstaining from brushing, microbial plaque lines the outer portion of the JE. Cells within the JE (Langerhan-type antigen presenting cells) sample members of dense multispecies indigenous flora that are pressed up against its periphery and move these components into subjacent lymphatic vessels. The broad ability of cells associated with innate immunity that display pattern recognition receptors (PRRs) are designed to identify pathogen associated molecular patterns (PAMPs) present on the surface of persistent microbial contaminants of the JE. This local subgingival biofilm results in intracellular signaling pathways causing gene transcription and responsiveness in the tissue underlying the JE (43). These antigen presenting cells then migrate to the regional lymph vessels. After presenting these antigens to lymphocytes in the regional lymph nodes, the lymphocytes become sensitized and ultimately make their way back from the node to the locus of infection, the sulcus, where they confront the marauding plaque bacteria. This movement to and fro is guided by cytokines and particularly by ICAM and VCAM receptors. Repopulation of sensitized cells can take anywhere from 7 to 14 days after bacterial contact with the JE (44).

These highly orchestrated events represent a skeletal description of the interaction of the innate and acquired immune responsiveness. However, prior to this series of events a more primitive response occurs. Here almost immediately after the microbial assault the bacterial components make their way to the underlying vasculature initiating the complement cascade and migration of polymorphonuclear leukocytes (PMNs) to the sulcus to protect against the bacterial onslaught. Thus, within 2–3 days after abstinence from oral hygiene, marauding bacteria are confronted by a wall of complement and PMNs that line the outer border of the sulcus (45).

### Molecular

Aggregation is a double-edged sword, on one hand it forms a shield and protects the biofilm mass from antimicrobial agents and other environmental challenges. However, on the other hand, aggregation limits the ability of the microbe to mobilize and move away from danger. Dispersin B (dspB) is a hexosaminidase that attacks hexosamine containing matrix polysaccharides that consist of a N-acetyl –D- glucosamine residues (46). dsp B was first discovered in *Aa* in 2003 by Kaplan et al. (47). Kaplan developed two dramatic experiments that exemplified how an organism whose main phenotype is aggregation could migrate and escape from its captivity (48). In the first experiment in an enlarged tissue culture dish, a single mother colony of *Aa* was carefully inoculated at one side of the plate and then 3 days later the daughter colonies had migrated away from the mother colony at what was calculated as a distance of over 2,500 human miles (47). The migration was due to the rupture of the biofilm membrane and the explosive projection of *Aa* to a distant site (48).

**Figure 4:**
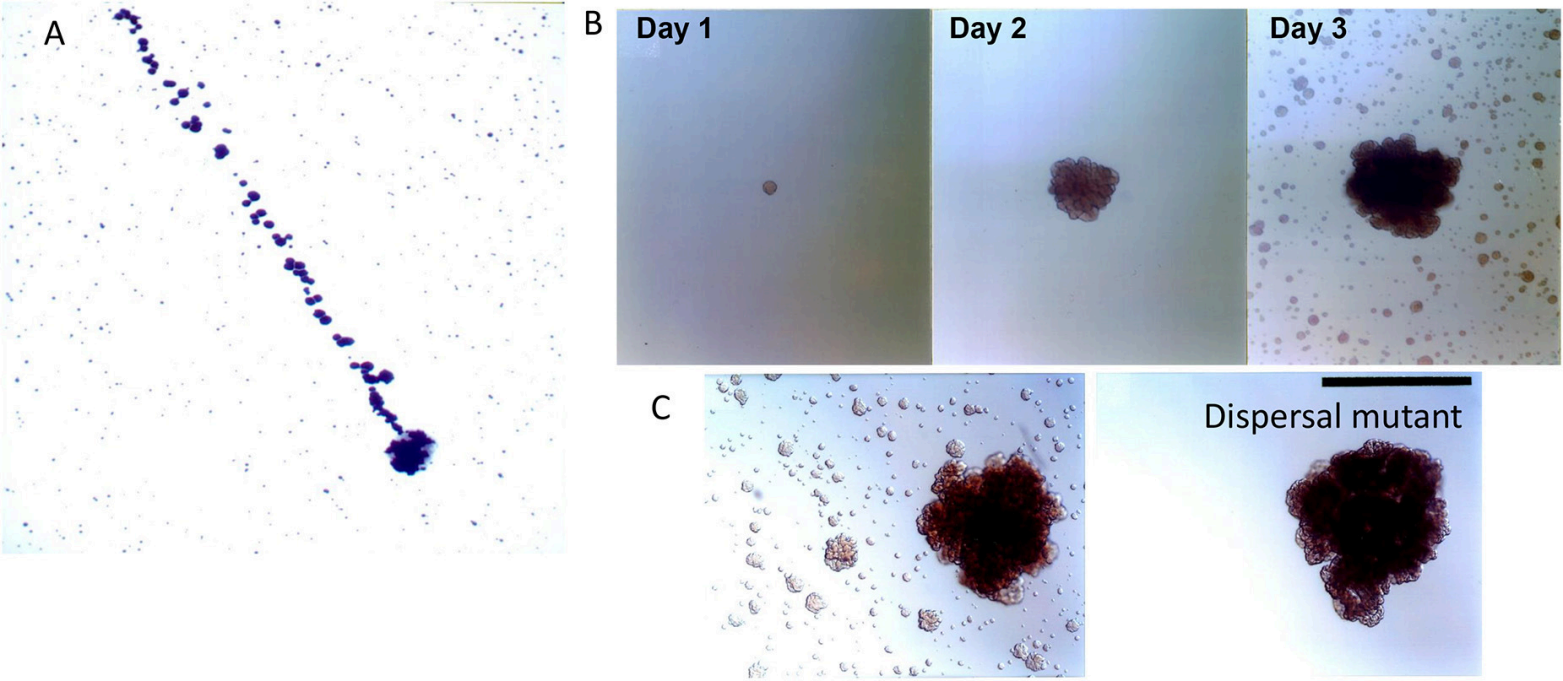
Biofilm dispersion by *Aa*. Illustration of dispersal of daughter colonies (top of panel **A**) derived from initial mother colony (bottom area of panel **A**). Distance covered by calculation of the 1 micron size of *Aa* is estimated at 2,500 human miles calculating the distance from location of *Aa* at bottom to location of *Aa* on top of panel **(A)** [Modified from Kaplan et al. (47)]. Depiction of colonies adhering to capillary pipette immersed in broth containing *Aa* after 1, 2, and 3 days of *Aa* incubation. Note the satellite colonies of *Aa* seen in broth surrounding capillary pipette after 3 days of growth **(B)**. Panel C depicting the wild type parental strain containing *dspB* as compared to the transposon mutant. Note that the mutation has affected the presence of satellite colonies as seen in right panel. Left panel shows wild type Aa expressing dispersen **(B)** and showing satellite colonies **(C)** [Modified from Kaplan et al. (49)].

In a second series of experiments *Aa* was allowed to grow on capillary pipettes immersed into a broth containing *Aa* (48). *Aa* colonized the pipette leaving the surrounding broth clear of cells. Three days later satellite colonies derived from those cells adhering to the pipette were found in the surrounding broth. Using transposon mutagenesis, the gene responsible for producing the satellite colonies was interrupted and identified as an enzyme called dispersin B (Figure 4). The fact that the enzymes had specificity for a polysaccharide substrate, PGA substantiated the finding. The 3 dimensional structure of the enzyme and the substrate it disrupted illustrated the process, its function, and how its discovery provided a new model of transportation and mobilization for *Aa* (48).

### Experimental

In a series of elegant experiments Stacy et al. illustrated the biological relevance of dspB. Using a dual species biofilm model consisting of *Aa* and *S. gordonii* it was demonstrated that lactate, the preferred carbon source for *Aa*, was provided by *S. gordonii* (50). Further *S. gordonii* was also responsible for producing H_2_O_2_ that could be toxic to *Aa*. Stacy et al. showed that in the presence of excessive levels of H_2_O_2_ *Aa* up-regulated genes such as; *katA*, responsible for catalase, which can neutralize H_2_O_2_ (50). However, in times when H_2_O_2_ was produced in excessive levels, *Aa* up-regulated *dspB* permitting *Aa* to move away (likely subgingivally) to avoid the toxic effects of H_2_O_2_. Thus, *dspB* provides a survival mechanism for *Aa* when it confronts environment stress (the Stacy experiments) or when it lives in an environment where nutritional stress occurs such as seen in the Kaplan experiments (48, 50).

### Conclusion

The discovery of *dspB* has provided a mechanism for mobility leading to protection. *Aa* has figured out how to have the best of both worlds; nutritional convenience by virtue of its close association with lactate producing species and locomotion away from products such as H_2_O_2_ formed by these same lactate producing species in order to avoid the hazardous conditions created by these species (Figure 4).

## Misconception 4: *Aa* is the Causative Agent in LAgP: Overcoming Host Restrictions (Suppressing Host Defenses)

### Clinical

Several early studies suggested that *Aa* was the causative agent responsible for LAgP. The association of *Aa* with disease was re-inforced by the fact that *Aa* possessed leukotoxin that killed white blood cells as well as other virulence attributes that gave biological plausibility to *Aa* as a causative agent. Several recent studies have been performed examining microbes related to the development and progression of LAgP (6, 51, 52). These studies attest to the relevance of *Aa* in this disease, however, its role with respect to causation is challenged (6) and although *Aa* is necessary for disease it needs partners. In the case where the more virulent higher leukotoxic *Aa* strain was found, the association of *Aa* with causation may be more relevant (51, 52). To the greatest extent early studies isolated plaque from previously diseased sites and therefore the cause and effect relationship was open to question (5). The first major study to examine subjects going from health to disease was a study showing that African American adolescents who carry *Aa* show a greater relationship to disease as it progresses (7). These findings were extended in studies by Haubek et al. who in another longitudinal study showed an increased relative risk for the JP2 strain (the hyper-leukotoxin –producing strain of *Aa*) although there was a much reduced relative risk shown for the non-JP2 strain (the moderate leukotoxic strain of *Aa*). These studies suffered from the fact that only *Aa* and no other microbe was examined in the plaque matrix.

The first longitudinal study to examine *Aa* relative to the overall commensal flora going from relative health to disease was done in 2013. This study was an observational longitudinal human study that was designed to detect progression from health to disease in African American or Hispanic adolescents (6). Of over one-thousand subjects screened, 63 *Aa*-positive and 72 *Aa*-negative students, who were initially periodontally healthy, were selected to follow for a 2–3 years observational period. Out of the 135 students enrolled in the longitudinal study 16 students and 18 sites developed bone loss over 2–3 years (6). Using the HOMIM technology 5 microbes were highly associated with disease prior to detection of bone loss. Of the 700 taxa followed, *Aa, S. parasanguinis* and *Filifactor alocis* were most highly related to sites that developed disease as compared to sites in the same individuals that had *Aa* and remained healthy. The association of this consortium occurred 6 months prior to bone loss with a sensitivity of 89% and a specificity of 99% (6). Cross-sectional studies examining subjects with LAgP, healthy housemates, and healthy others, also from African American backgrounds found similar constellations of microbes (53). Other cross-sectional studies from other ethnicities have found distinctly different combinations of microorganisms (54). The association of *Aa* with *S. parasanguinis* is consistent with the findings of the Whiteley group and supports the concept of nutritional interdependency since *S. gordonii* and *S. parasanguinis* provide lactate and have similar physiological properties (35). The 2013 study is the first report of such an association in the subgingival region of a site that was healthy and developed aggressive periodontitis.

### Molecular

Leukotoxin (Ltx) was discovered in 1977 by Taichman and was first biochemically characterized by Baehni et al. (55) and Tsai et al. (56) as a toxin that kills leukocytes and lymphocytes (55–57). The genetics of Ltx was determined almost simultaneously by Lally et al. and Kolodrubetz et al. in (58, 59) and the difference between the minimally toxic and highly toxic strains were illustrated in clinical studies by Haubek et al. (51). The fact that the toxin was both secreted and membrane bound was first determined by Kachlany et al. in (60). The importance of the promoter region in elevated toxin production is still ongoing (61). Thus far, all bacteria that show this toxin have been implicated in disease, particularly infectious disease caused by a multispecies consortium (27). It is our belief that the toxin neutralizes the local mucosal immune response to enable other bacteria to overgrow.

ApiA was first discovered in 1999 as one of six Outer Membrane Proteins [OMPs] (44) thought to play some role in the pathogenesis of Aggressive Periodontitis. Initially ApiA was called OMP 29 and then Omp 34 when heat treated (62). However, it appears as if this 100 mw OMP autotransporter exists as a trimer that has multiple functions. The phenotypic characteristics of ApiA include; adhesion, invasion, and complement resistance. As for complement resistance, based on the work of Asakawa et al. it appears as if factor H binding occurs somewhere between 100 and 200 amino acids in the 295 ApiA amino acid protein (63). While invasion and adhesion appear to occur in separate regions of the protein this trimeric autotransporter/multifunctional protein plays a significant role in *Aa*'s survival and immune resistance as demonstrated by its upregulation during environmental stress and turbulence (as described below).

### Experimental

While all studies do not support the role of *Aa* in LAgP almost all studies of adolescents of African or Middle Eastern descent do support this observation (64). Moreover, studies that do claim a role for *Aa* suggest that the JP2 type hyper-leukotoxin producing strain is more virulent (65). The most likely explanation for these discrepancies can be placed on geographic, ethnic, and genetic differences in susceptibility and in exposure to bacteria. However, relative to populations exposed and sensitive to *Aa*, we are much closer to understanding how the microbe affects the disease process as follows.

Shortly after plaque development changes occur in the JE. These changes make the JE more vulnerable to penetration by microbial antigens that border the basement membrane creating a standing osmotic gradient that causes changes in the connective tissue vasculature subjacent to the bacterial/epithelial barrier. Sampling of these antigens by antigen presenting cells causes stagnation of blood flow in these vessels, leukocyte margination, pavementing of leukocytes, and then diapedesis due to chemotactic signals guiding PMNs to mobilize in the direction of the overwhelming microbial burden bordering the epithelial barrier (45).

The first element that confronts this microbial burden is a serum exudate that consists of complement and PMNs that can kill bacteria either directly or indirectly. Complement appears to act directly on the cell wall of bacteria, punching holes in the membrane resulting in cell lysis (66). PMNs can engulf and degrade microbes at a rapid rate. *Aa* is equipped with strategies to neutralize these two potent host innate defense systems. Not only does *Aa* possess ApiA, a complement effector molecule, and leukotoxin, a toxin that kills PMNs, but it has been shown that, under stress, these genes are up-regulated as a defense against these host elements (67).

In the experimental model described below the association of the up-regulation of Ltx expression when *Aa* is under stress was shown in a model that examined an erythromycin resistant strain of *Aa* challenged by high doses of erythromycin [Er] (67). The Er challenged cells showed increased biofilm formation and up-regulation of 4 genes; *flp, pga, ltx, and tfox*, in the Er resistant *Aa* strain. This up-regulation showed an unexpected but previously demonstrated linkage between *ltx, pga* and *flp* expression when *Aa* was under stress (67).

Two host innate elements that appear to be related to disease susceptibility in studies of limited but well-defined subjects with LAgP are lactoferrin and PMN functionality (68, 69). Thus, both lactoferrin (Lf) and PMN differences have been observed in African American adolescents (69, 70). In the case of Lf, a single nucleotide polymorphism in the N-terminal- antimicrobial region of Lf appears to have an impact on oral microbial constituents aside from *Aa* in these populations (71). This Lf activity could potentially increase vulnerability to LAgP by altering microbial growth and survival although susceptibility appears to vary based on ethnicity (71). Further, these adolescents appear to have Lf containing minimal iron that permits *Aa* to colonize with greater efficiency as compared to controls who have Lf with high iron content (72). With respect to PMNs, reduced chemotaxis appears to be due to primed PMNs resulting in hyperactive PMN responsiveness, impaired phagocytosis, and overproduction of superoxide (73). In combination these alterations in innate immunity could potentiate disease susceptibility.

### Conclusion

**Figure 5:**
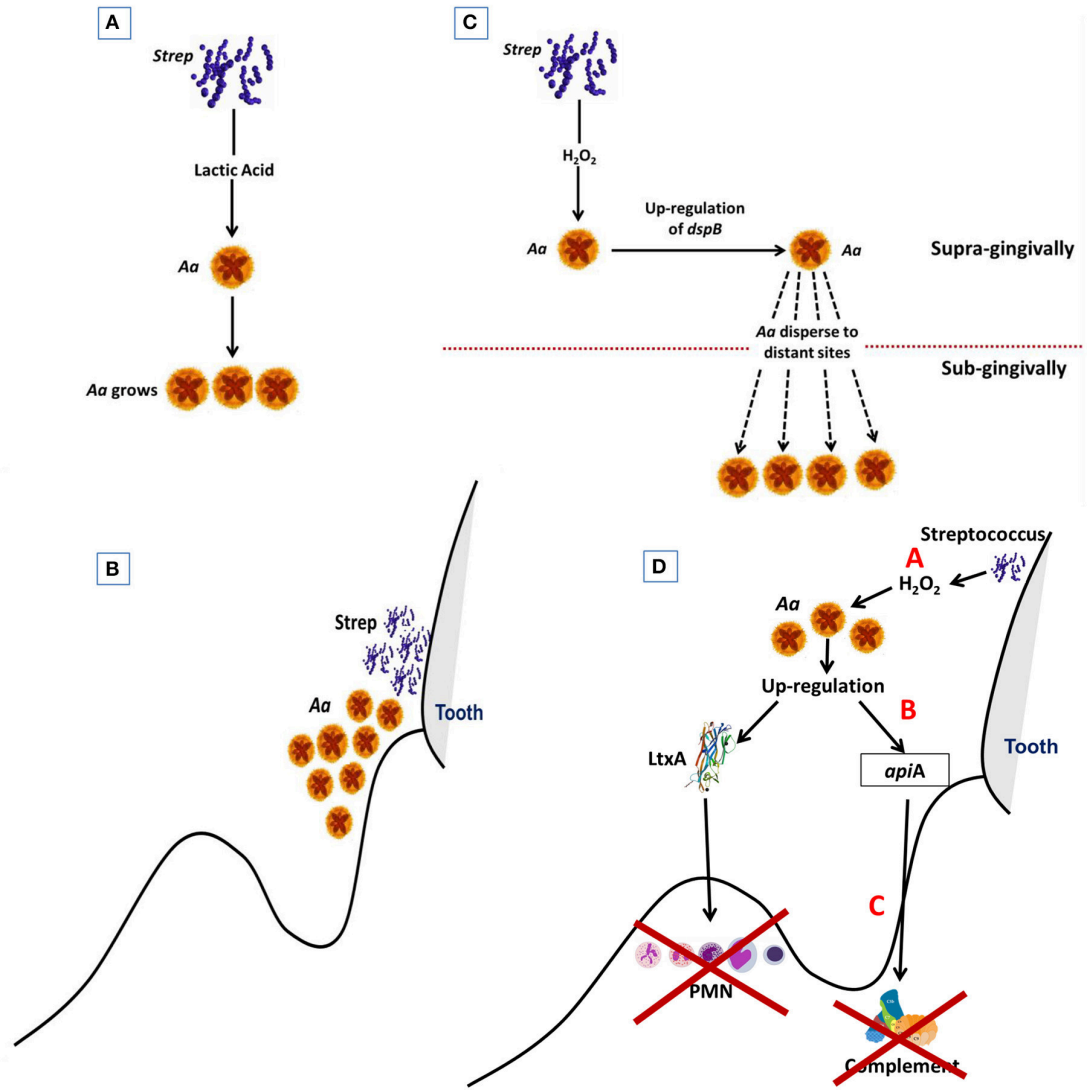
Diagram Illustrating steps by which Aa is activated to subvert host defense. Streptococci supplies Aa with lactate in early steps in colonization process. This helps *Aa* attach to the native tooth surface (Step 1; **A**). Streptococci and Aa partner. Streptococci provide Aa with lactate that helps *Aa* survive after binding to tooth surface (Step 2; **B**). Excessive amounts of peroxide produced by streptococci causes stress for *Aa* which results in upregulation of *dispB* which causes *Aa* to migrate away from peroxide to subgingival area (Step 3; **C**). Stress also causes upregulation of Leukotoxin (Ltx) and ApiA. Ltx blunts the initial PMN response and ApiA causes complement resistance. These two factors subvert the local host response and allow other microbes to overgrow due to dysbiosis of the host local environment. (Step 4; **D**).

Early in the disease process *Aa* is confronted with potent host defense systems consisting of a wall of PMNs and a high concentration of complement, both of these host defense elements can destroy *Aa* and other potential pathobionts. Alteration of these host defense systems could permit *Aa* to survive. Nevertheless, these defense systems while altered still exist and put stress on *Aa* and other members of the flora. Responding to the stress *Aa* produces agents that can neutralize host defenses to enable other microbes in the region to survive and as such acts as a useful participant in local host dysbiosis (Figure 5). In this respect *Aa* could be designated as a keystone pathogen (74).

## Overall Conclusions

Molecular and experimental models conclusively demonstrate that *Aa* is an early colonizer, co-colonizing with other early colonizers who produce lactate. *Aa* can then migrate away from stressful challenges to a more protected subgingival domain by up-regulation of *dspB*. Facing the challenge of an innate subgingival response, *Aa* can up-regulate complement resistance genes and leukotoxin production to modulate the local host immune response in order to allow for an overgrowth of a consortium of pathobionts. Working together the consortium can overwhelm the natural resistance of the local host innate defense response and produce inflammatory cytokines that can result in connective tissue and bone loss and derangement of the attachment apparatus. It is still to be proven whether this scenario is unique to the non-JP2 strain of *Aa* or if this is a general strategy used to provoke local disease. This working hypothesis can be applied to other combinations of microbes that can operate under differing circumstances in populations distinct from the African American adolescents studied in Localized Aggressive Periodontitis.

## Author Contributions

All authors listed have made a substantial, direct and intellectual contribution to the work, and approved it for publication.

### Conflict of Interest Statement

The authors declare that the research was conducted in the absence of any commercial or financial relationships that could be construed as a potential conflict of interest.
